# The roles of implicit approach motivation and explicit reward in excessive and problematic use of social networking sites

**DOI:** 10.1371/journal.pone.0264738

**Published:** 2022-03-16

**Authors:** Michael Wadsley, Niklas Ihssen

**Affiliations:** Department of Psychology, Durham University, Durham, United Kingdom; Charles Darwin University, AUSTRALIA

## Abstract

Despite growing concerns about the addictive potential of social networking sites (SNSs), little is known about the precise neural, cognitive, and emotional processes underpinning compulsive SNS behaviours, such as excessive checking of SNSs. Recent evidence points to the important role of reward in SNS behaviours and one avenue to examine reward processes related to SNSs is the use of behavioural paradigms that allow for the measurement of implicit motivational responses, such as the approach avoidance task (AAT). The AAT has been successfully utilised to capture changes in unconscious reward processes in substance use disorders and other behavioural addictions, with faster approach reactions to addiction-related stimuli reflecting increased wanting/urges to have/consume the reward. In the present study 411 young adults completed an online Visual Approach/Avoidance by the Self Task (VAAST) with social media and control logos as well as other subjective (explicit) measures of reward experience related to SNSs. Our results showed that across participants SNS logos elicited strong approach reactions (compared to control stimuli) and that stronger SNS approach tendencies predicted more frequent SNS checking. Importantly, increased approach motivation was not associated with more problematic use. However, both checking frequency and problematic use were related to alterations of explicit reward processing, including the subjective experience of SNS urges or wanting. We conclude that changes in automatic approach motivation towards SNS stimuli are common in most SNS users, which suggests that implicit imbuement of social media with reward has become pervasive among young adults. Problematic SNS use however may be more reliably indicated by changes in explicit reward processing, such as subjective wanting.

## Introduction

Despite a burgeoning literature purporting to demonstrate the addictive potential of social networking sites (SNSs) and describing behavioural patterns of “problematic” or “compulsive” SNS use, the formal recognition of SNS addiction as a mental disorder remains controversial [1, 2]. Many studies are limited by their atheoretical approach and do not offer an explanation of the conditions under which compulsive SNS use develops or how it is maintained [3]. Nevertheless, a growing body of research has begun to employ behavioural experimental paradigms to examine the cognitive and motivational processes (such as inhibitory control and attentional bias) that might contribute to general SNS use behaviours and the development and maintenance of problematic SNS use [e.g., 4–6]. If problematic SNS use is to be considered a candidate behavioural addiction then such studies investigating the cognitive/motivational components of SNS use are important in order to demonstrate that it warrants recognition as an addictive disorder [7].

Recently, we have proposed that adopting a reward and incentive-sensitisation approach to understanding SNS use and its addictive potential may be of substantial use to researchers [8]. The incentive-sensitisation theory of addiction emphasises a dissociation between drug liking and drug wanting, whereby repeated drug use leads to sensitisation of the brain’s *wanting* system, rendering it hyper-reactive to the drug and drug associated stimuli [9]. This happens without concurrent change to the brain’s *liking* system, explaining how addicts can develop increasingly compulsive urges to take the drug without experiencing increased pleasure from drug consumption. While the theory was originally proposed to explain substance use disorders, evidence now also exists that these mechanisms may extend to addictive behaviours [10]. Thus, dopamine dysregulation resulting from the rewarding aspects of SNS use may produce intense *wanting* without *liking* that gives rise to compulsive urges to use SNSs and potentially addiction [8]. As such, research that can demonstrate modifications of *wanting* in excessive and problematic SNS users may provide key evidence in support of its addictive potential. In our previous study [8] we have presented preliminary data showing that subjectively reported wanting co-varies with the frequency users check their SNSs and with diagnostic markers of problematic SNS use. Importantly, according to the incentive-sensitisation theory and its associated incentive salience model [e.g., 11], wanting processes (and its dysregulation in addiction) do not only manifest at a conscious subjective level (i.e., in explicit urges and desires) but also entail “implicit”, unconscious changes of motivation. Such an account is also upheld by dual-process models of addiction that view addictive behaviours as being driven by both an “impulsive” system, responsible for the automatic evaluation of stimuli, and a “reflective” system, responsible for more controlled and deliberate processing that enables self-regulation of behaviour [12–15]. Thus, addictive behaviour can be understood as the outcome of an imbalance between implicit and explicit cognitions that results in a more reactive impulsive system (e.g., modified RTs to addiction-related cues), and a reflective system that is less able to voluntarily inhibit the behaviour or regulate emotional responses (e.g., self-reports of increased subjective craving). Furthermore, explicit and implicit cognitions have been shown to uniquely predict substance use behaviours, underscoring the utility of assessing both the conscious and unconscious mechanisms underlying addictive disorders [16, 17].

The present study set out to identify markers of implicit wanting in excessive and problematic SNS users by employing an approach-avoidance task. A second objective was to extend our previous study by systematically measuring subjective (explicit) reward responses using a large sample of SNS users and a range of established reward paradigms, including visual cue reactivity, reward choice, and self-reports of wanting/liking. Demonstrating a distinct profile of behavioural responses in these tasks in excessive/problematic users would add further weight to the idea of SNS use having an addictive dimension.

### Implicit wanting and the approach-avoidance task

One way to demonstrate incentive salience (and incentive-sensitisation) in humans is to employ behavioural tasks that capture changes in attention during exposure to visual stimuli associated with the reward (e.g., pictures of the drug). During the course into addiction, these cues can become associated with the rewarding effects of the drug (or behaviour) and imbued with heightened incentive salience, increasing the likelihood that they will attract attention and create an “attentional bias” [18, 19]. Two frequently used measures of attentional bias in the laboratory are the Dot Probe task [20] and visual search task [21]. While such paradigms have received substantial interest in drug addiction research [22, 23] and are increasingly employed to investigate potential behavioural addictions [24, 25], studies examining attentional bias in excessive SNS users have reported conflicting results. Nikolaidou et al. [6] used eye-tracking whilst participants completed a Dot Probe task with SNS-related stimuli and matched controls. They found that compared to non-problematic SNS users the gaze of problematic users dwelled significantly longer on SNS-related stimuli compared to non-computer related control stimuli and this effect occurred without problematic users rating the SNS images as more pleasant. However, when employing a visual search task Johannes et al. [4] found no impairment in performance when SNS logos (either with or without notification signs) were used as distractors compared to non-social iPhone app controls. In line with this study but using a novel and more ecologically valid visual search task, Thomson et al. [26] found no evidence that an attentional bias to SNS logos is correlated with addiction severity. However, while measures of attentional bias have so far not shown clear evidence of altered attentional processing of SNS cues (in both “regular” and compulsive users), these cues might still elicit stronger motivational responses than control cues and/or differentiate motivational responses of regular users from those of compulsive users.

One task to investigate (implicit) motivational responses directly is the approach-avoidance task (AAT; [27]). The AAT is a behavioural reaction time task that measures automatic approach and avoidance tendencies by presenting pictures which participants are instructed to either approach (move or pull towards) or avoid (move or push away from) using joystick push/pull (arm extension/flexion) movements or variants thereof. As motor approach behaviour towards conditioned reward cues directly reflects the motivation to engage or have the reward [11], the AAT allows to measure wanting implicitly. Researchers have previously employed modified versions of this task to investigate changes in implicit wanting associated with alcohol misuse [28, 29], problem gambling [30] and food rewards [31, 32]. Yet, we are currently aware of only one study that has utilized this paradigm to investigate problematic SNS use. Juergensen and Leckfor [33] used an AAT with Facebook related icons (e.g., Facebook app icon, like button, friend request icon) and neutral control stimuli (e.g., unfamiliar SNS icons) and found that greater scores on a measure of Facebook addiction were correlated with stronger approach biases for SNS stimuli but not control stimuli. Thus, participants who self-reported more problematic Facebook use were more likely to display faster approach reactions to Facebook-related stimuli.

More recently, a conceptually similar task, the stimulus response compatibility task (SRC task; [34, 35]) has been employed in a study using Facebook (vs. control) cues to measure implicit approach and avoidance reactions [36]. In this task participants make symbolic approach or avoidance movements to addiction-related or control stimuli by moving a manikin figure towards or away from the image (typically using keyboard responses). In contrast to Juergensen and Leckfor [33] the results of Du et al. [36] indicate a general approach bias for Facebook stimuli in the whole sample of Facebook users, however this effect was unrelated to Facebook self-control failures (i.e., using social media when it conflicts with other goals). Critically, so far the studies that measure automatic SNS-related approach and avoidance reactions have employed a modest sample size [cf. 33] and/or exclusively used Facebook-related cues. Thus, while the measurement of automatic approach and avoidance tendencies appears to be a promising tool to demonstrate implicit approach motivation in substance-related and other behavioural addictions, more systematic work is needed to ascertain whether these effects reliably translate to the problematic use of SNSs.

In the present study we sought to investigate this issue by measuring implicit approach responses to SNS cues in a well-powered sample of 411 young adults. To facilitate online testing we employed an adapted version of the Visual Approach/Avoidance by the Self Task (VAAST) which has been shown to produce larger compatibility effects than the SRC (Manikin) task [37]. Previous AATs have relied on push/pull movements (e.g., using joysticks) to infer automatic approach/avoidance tendencies, however Rougier et al. [37] have challenged the necessity of these motor processes. Instead, the VAAST simulates the visual information associated with whole body movements by enlarging or reducing the size of an image (depending on button-press responses), giving the perception of an approach or avoidance movement.

### Explicit motivational responses to SNS stimuli

Importantly, apart from the AAT, we also included three measures of explicit motivational (reward) responses in our study. First, we included a measure of visual cue reactivity to establish whether social media logos with notification icons induce explicit wanting or craving of SNS use as a function of usage frequency or problematic use. While the cue reactivity paradigm is often used to probe implicit reward processes (e.g., neural or physiological responses), cue exposure can also be used to measure variations in explicit motivation, such as self-reported wanting urges assessed in our study. Our previous preliminary findings suggested a positive association between cue reactivity and usage frequency/problematic use [8] but were limited by the lack of control/baseline measures–which we include in the present study–to account for differences in general trait levels of wanting. Secondly, and similarly to our previous study, we incorporated questionnaire-based measures of SNS liking (pleasure) vs. wanting (motivation). Following the incentive-sensitisation theory [9, 10], we aimed to establish a potential dissociation between these measures in predicting excessive and problematic SNS uses.

Finally, we used a reward choice task in which participants were asked to decide between a variable monetary reward and a SNS reward (obtaining likes). Choice tasks are a cornerstone in animal research on reward, offering an objective readout of the motivational value attributed to a stimulus relative to an alternative [e.g., 38]. They have also been used in various versions with humans (e.g., choice between different food rewards, [39]).

Mirroring findings in substance addiction, we predicted that (1) the difference between the approach RTs to SNS logos and approach RTs to control logos was negatively associated with frequency of checking and problematic usage (i.e., faster SNS approach tendencies predicted more excessive/problematic usage); (2) the difference between visual cue reactivity to SNS versus control logos was larger in individuals with more frequent checking and larger in individuals with more problematic usage; and (3) self-reported wanting but not pleasure/liking of social media use was positively related to frequency of checking and problematic usage. Data from the reward choice task and other secondary measures (see below) were examined in exploratory analyses. All hypotheses and analyses were preregistered on the Open Science Framework website (https://osf.io/jqm57).

## Method

### Participants

The link to the online study was accessed 466 times. Only responses from individuals who participated in the study to completion were included in the analysis. This resulted in a final sample of 411 participants (190 male, 214 female, 7 other) aged between 18–30 (M = 22.9, SD = 3.55) who completed the study between April—July 2020. An international sample was recruited, the majority of which were students (61.6%) and the most common nationality was British (21.4%). Most of the recruitment (69.4%) took place through Polific.co and SurveySwap.io, with the remaining participants (30.6%) being recruited through internal channels at Durham University or by other means. Participants were either reimbursed with small monetary incentives or course credits for their participation or took part without reimbursement. The study was approved by the Ethics Sub-Committee in the Department of Psychology at Durham University and all participants provided fully informed consent.

### Materials

#### Implicit reward measure (approach/avoidance tendencies)

**Fig 1:**
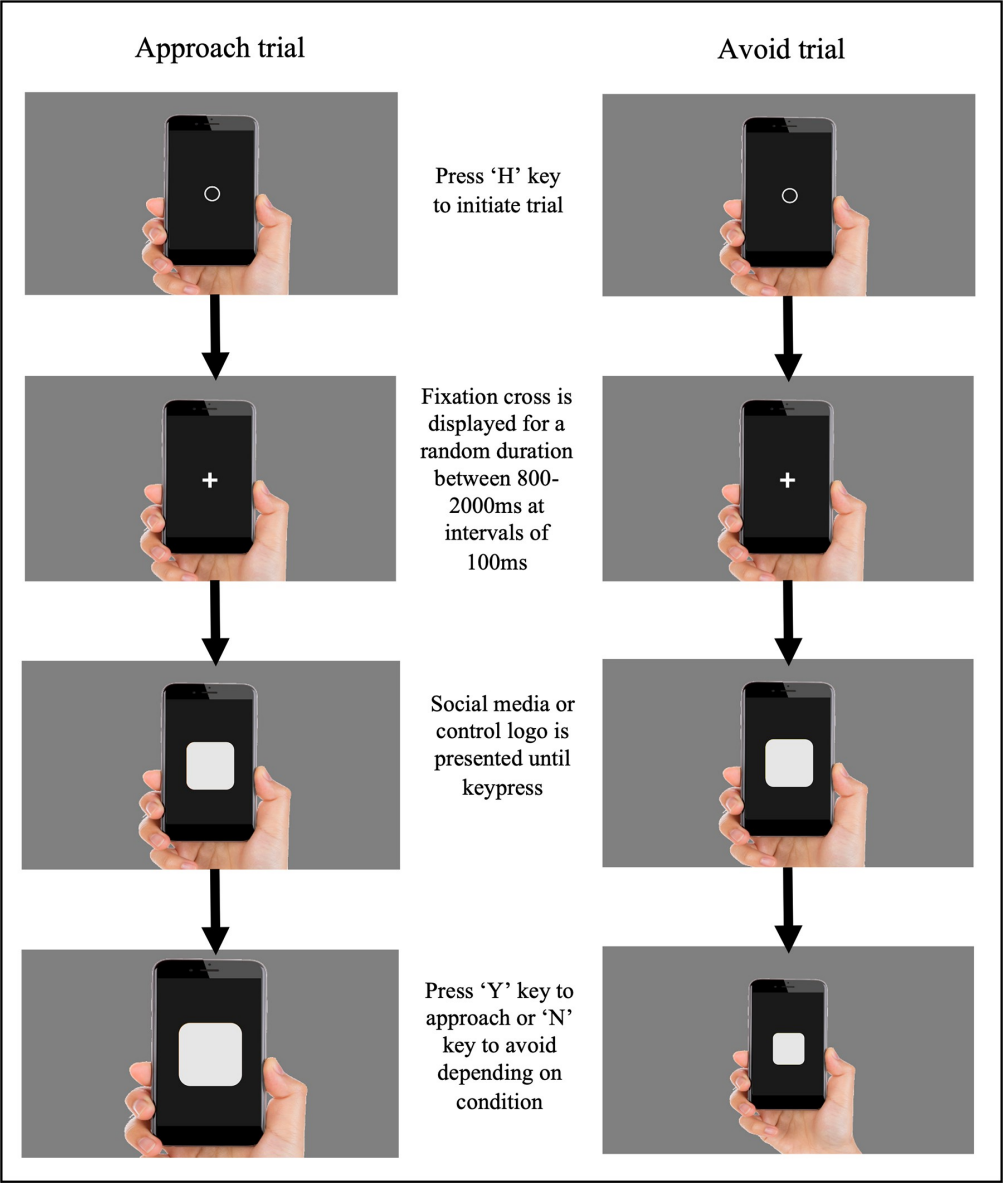
Example of the trial sequence in the social media VAAST with an approach instruction (left) and avoid instruction (right). Social media and control app logos have been removed for copyright reasons.

Participants completed a Visual Approach/Avoidance by the Self Task (VAAST; [37]) with SNS logos and matched control stimuli. Online versions of the VAAST using PsyToolkit have previously been shown to be a reliable measure of approach/avoidance tendencies and produce effects that are of a similar magnitude to those obtained in lab versions of the task [40]. In this task participants were required to move towards or away from SNS or control logos using the computer keyboard. Participants were instructed to approach SNS logos and avoid control logos in the first block, and vice versa for the second block, while others completed the same blocks in the reversed order (randomised across participants). SNS stimuli consisted of Facebook, Instagram, Twitter, Snapchat and YouTube logos. Control stimuli consisted of 5 iPhone app icons (Maps, Weather, App Store, Books and Photos). A training phase with feedback occurred before the experimental trials in each block. The training phase consisted of 10 trials where every stimulus was presented once in a random order (i.e., 5 social media and 5 control stimuli). During each trial an image of a hand holding a smartphone was displayed on a grey background. Participants pressed the ’H’ key to initiate the trial. A fixation cross was shown in the middle of the screen for a random duration between 800-2000ms at intervals of 100ms. This was followed by the stimulus presentation (i.e., SNS or control app icon overlayed on the smartphone screen) which remained on screen until a response was made. Participants pressed the ’Y’ key to approach or the ’N’ key to avoid, depending on the condition. As a result of the key press the hand holding a smartphone and app icon either increased (approach) or decreased (avoid) in size, giving the appearance of movement (see Fig 1 for an illustration of the trial sequence). An inter-stimulus-interval of 500ms occurred after each response. Incorrect responses consisted of making approach responses (i.e., pressing the ‘Y’ key) on avoidance trials, making avoidance responses (i.e., pressing the ‘N’ key) on approach trials, or pressing the ‘H’ key on either trial. During the practice trials an error message was displayed after incorrect responses. No feedback was given about the accuracy of responses during the experimental trials. In the experimental phase a total of 40 trials occurred in random order for each block. Participants completed a total of 100 trials (including blocks of 2 × 40 experimental trials and 2 × 10 training trials).

#### Explicit reward measures

*Cue reactivity*. Participants were shown the logos of different social media platforms including Facebook, Instagram, Snapchat, Twitter and YouTube. Participants were only presented with those social media that they reported having used in the previous week. Each logo appeared with a red notification icon in the top right corner of the logo, containing the number one. For each logo shown, participants were asked to report the extent to which the image made them want to check the corresponding social media account. Responses were made using a 5-item Likert-scale (1 = disagree, 2 = slightly disagree, 3 = neither agree nor disagree, 4 = slightly agree, 5 = agree). In addition, participants were presented with two control logos consisting of either the App Store or Play Store logo (depending on whether they reported using an iPhone or Android) and the BBC iPlayer logo. As before, participants rated the extent to which these logos made them want to check the corresponding app using a 5-item Likert-scale. A cue reactivity score was calculated for each participant by subtracting the average response to control logos from the average response to the social media logos.

*Wanting vs*. *liking*. Participants were asked to respond to four statements that assessed the extent to which they enjoyed using social media (e.g., “Using social media is an activity that gives me pleasure”), and four statements that assessed the extent to which they experience wanting or urges to use social media (e.g., “When I see my phone, I experience a strong desire to check social media”). Participants indicated their agreement with each statement using a 5-item Likert-scale. Scores across the four wanting measures and the four liking measures were averaged to produce overall wanting and liking scores for each participant.

*Money-or-likes*. Participants reported the average number of “likes” or “retweets” they would expect to receive on a typical SNS post. They also completed an experimental reward choice task, in which they first indicated their preference for receiving either £1 or double the likes on their next social media post. When participants chose the social media reward the question was repeated with incremental increases in the value of the money reward, with the increments approximating a doubling of the previous value (i.e., £2, £5, £10, £25, £50, £100). Accordingly, the point at which participants discontinued incrementing the monetary value was interpreted as the subjective reward strength of receiving likes on an SNS post, with higher monetary values representing stronger reward.

#### Other variables

*Excessive and problematic SNS use*. Two different measures of excessive SNS use assessed (a) self-reported daily time spent using SNSs (hours), and (b) the frequency of checking using a 7-item scale (less than daily, daily, every 3-5h, 2h, 1h, 30mins, 15mins). Participants were asked to provide separate estimates of their usage intensity before and after the COVID-19 virus outbreak. Participants were also prompted to use the screen time app on their phone (where possible) for questions related to their current usage to enable more accurate estimates. As the present study was more interested in current usage behaviour, only the estimates relating to participant’s more recent usage intensity (post-COVID-19 outbreak) are reported.

Problematic SNS use was assessed using the Social Media Disorder Scale (SMDS; [41]). The scale measures nine proposed criteria for SNS addiction (Preoccupation, Tolerance, Withdrawal, Persistence, Displacement, Problem, Deception, Escape, Conflict) using a dichotomous yes-no response scale. The level of problematic use was calculated for each participant by summing affirmative responses across all items (scores could range from 0–9). It is suggested that a score of 5 or more on the SMDS indicates an SNS addiction. Scores were not calculated for participants who had missing data on one or more items in the scale.

*General sensitivity to reward*. Participants responded to 10 questions taken from the SPSRQ-20 [42], a short form of the Sensitivity to Punishment and Sensitivity to Reward Questionnaire [43]. Only the questions relating to general sensitivity to reward were used (e.g., ‘Does the good prospect of obtaining money motivate you strongly to do some things?’). Participants responded to each item using a dichotomous yes-no scale. Scores across the 10 questions were summed to produce an overall sensitivity to reward score for each participant. Scores were not calculated for participants with missing data.

*Self-awareness of problematic use*. At the end of the study participants were asked to respond to two statements relating to their own perception of their social media use: “I believe I use social media too much” and “I believe I am addicted to social media”. Participants responded to each statement using a dichotomous yes-no scale.

### Procedure

Participants who met the eligibility criteria (between the ages of 18–30 years old and with normal or corrected-to-normal vision) were invited to take part in an online study programmed using PsyToolkit [44, 45]. The experimental and questionnaire measures reported in this article were embedded in a larger online study that also investigated different motives underlying SNS use. These data were not directly relevant to the current research questions and have been reported elsewhere [46]. All participants accessed the study on a device with a real keyboard and using a browser other than Safari (for compatibility with the experiments coded on PsyToolkit). The average time taken to complete the study was 20.09 minutes. The data which support this publication are available on https://doi.org/10.17605/osf.io/dkr9q.

## Results

### Descriptive statistics

The most frequently used SNS in our sample was YouTube (86.4%), followed by Facebook (84.2%), Instagram (83.5%), Twitter (50.1%) and Snapchat (37.5%). Other types of SNSs that were not explicitly assessed but which participants reported using included Reddit (13.1%), TikTok (7.3%) and Tumblr (3.2%). Only two participants (0.5%) reported that they did not use a SNS of any kind.

When asked to estimate their current social media usage intensity to the nearest hour, participants reported spending an average of 4.57 hours (SD = 2.79) on SNSs each day. The median frequency of checking social media was 6 (i.e., every 30 mins), and the mean SMDS score was 2.01 (SD = 1.64). Thirteen participants were excluded from the calculation of the mean SMDS score and one participant was excluded from the mean time spent using due to missing data.

Responses to the ‘self-awareness of problematic use’ measures revealed that 60.8% of participants believed that they used social media too much (1.2% did not respond). Furthermore, 29.9% of participants believed that they were addicted to social media (0.7% did not respond). According to van den Eijnden et al. [41] (and also in accordance with the cut-off point for internet gaming disorder proposed in the DSM-5) a score of five or more (out of nine) on the SMDS is required to be diagnosed as a disordered social media user. Examination of SMDS scores in our sample revealed that only 6.2% of participants met these criteria, suggesting that participants over-diagnose themselves as addicted.

### Preregistered analysis

#### Approach avoidance tendencies

Only RTs of correct responses were included in the analysis. RTs of correct responses that were below 200ms or above 3000ms were removed as outliers (see https://osf.io/jqm57), resulting in the exclusion of 0.68% of the trials. Participants with an accuracy rate less than 60% on either experimental block were excluded from the analysis. This resulted in the exclusion of 21 participants from the analysis. The overall accuracy rate of participants included in the analysis was 96.55% (SD = 3.78).

**Fig 2:**
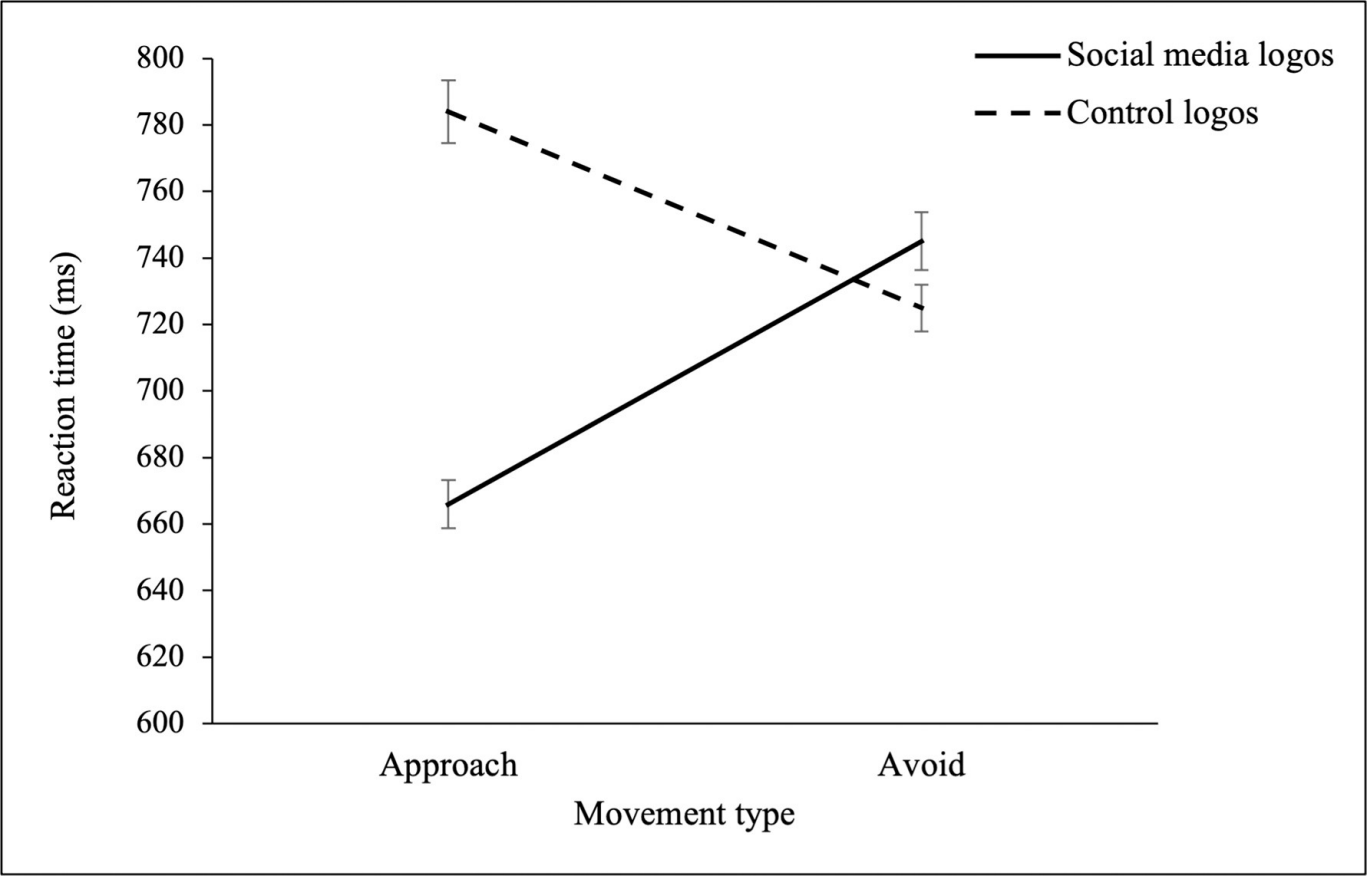
Mean approach and avoidance RTs (ms) to social media and control logos. Error bars represent mean SE.

First, the main effects of stimulus type (SNS vs. control logo) and movement (approach vs. avoid) as well as their interaction were analysed using a (non-preregistered) 2 × 2 repeated measures ANOVA. This revealed a significant main effect of stimulus type [*F*(389) = 184.95, *p* < .001, *η*_p_^2^ = 0.322], whereby participants responded faster to SNS stimuli (M = 706, SD = 157) compared to control stimuli (M = 754, SD = 162). The main effect of movement was also significant [*F*(389) = 7.69, *p* = .006, *η*_p_^2^ = 0.019], and revealed that participants were faster at making approach responses (M = 725, SD = 165) compared to avoidance responses (M = 735, SD = 155). Finally the interaction effect was also shown to be significant [*F*(389) = 99.38, *p* < .001, *η*_p_^2^ = 0.203], and this effect was further explored using (preregistered) paired t-tests. As shown by paired t-tests, mean approach RTs (ms) to SNS stimuli (M = 666, SD = 142) were significantly faster than approach RTs to control stimuli (M = 784, SD = 187), suggesting that SNS cues elicit speeded approach reactions [*t*(389) = -14.74, *p* < .001, *d* = 0.75]. In contrast, avoidance RTs to SNS stimuli (M = 745, SD = 172) were significantly slower than avoidance RTs to control stimuli (M = 725, SD = 137), indicating that social media cues produce more slowed avoidance reactions [*t*(389) = 2.71, *p* = .007, *d* = 0.14]. The mean RTs for each condition are displayed in Fig 2.

Accuracy rates were also compared using a (non-preregistered) 2 × 2 repeated measures ANOVA. The main effects of both stimulus type [*F*(389) = 36.95, *p* < .001, *η*_p_^2^ = 0.087] and movement [*F*(389) = 52.54, *p* < .001, *η*_p_^2^ = 0.119] were shown to be significant, revealing that participants were more accurate when responding to control logos and when making approach responses. The interaction effect was also significant [*F*(389) = 26.80, *p* < .001, *η*_p_^2^ = 0.064] and was further explored using paired t-tests. This revealed a significant difference between the accuracy on SNS approach trials (M = 0.96, SD = 0.06) and control approach trials (M = 0.99, SD = 0.03), indicating less accurate approach responses to SNS cues [*t*(389) = -9.70, *p* < .001, *d* = 0.49]. However, the difference in accuracy between SNS avoidance trials (M = 0.96, SD = 0.06) and control avoidance trials (M = 0.96, SD = 0.07) was nonsignificant [*t*(389) = -0.32, *p* = .750, *d* = 0.02].

Overall SNS approach tendencies were calculated for each participant by subtracting mean RTs on control approach trials from mean RTs on SNS approach trials. Similarly, overall SNS avoidance tendencies were also calculated for each participant by subtracting mean RTs on control avoidance trials from mean RTs on SNS avoidance trials. Approach and avoidance tendencies were negatively correlated with each other (*r* = -.58, *p* < .001) indicating that participants who were quicker to approach SNS stimuli were also slower to avoid SNS stimuli, when compared to control stimuli. SNS approach and avoidance tendencies were entered into three separate two stage hierarchical regressions to evaluate their unique contribution to time spent using, frequency of checking and problematic SNS use. For each regression approach tendencies were entered at stage one and avoidance tendencies at stage two. Results of the regression analyses are displayed in Table 1. For the regressions predicting time spent using and problematic use, SNS approach tendencies were not a significant predictor. Additionally, the inclusion of SNS avoidance tendencies did not provide a significant contribution to either model. However, SNS approach tendencies did significantly predict checking frequency (β = -.127, *p* = .012), whereas the inclusion of SNS avoidance tendencies did not provide a significant contribution to the model. Furthermore, the contribution of SNS approach tendencies remained significant after the inclusion of SNS avoidance tendencies (β = -.132, *p* = .034). Thus, while we observed a large SNS approach bias and a smaller but significant avoidance bias within the sample as a whole, stronger SNS approach tendencies did not predict prolonged or problematic SNS use. However, our results indicate that stronger SNS approach tendencies predict more frequent SNS checking to some small extent.

**Table 1:** Summary of regression analyses for the prediction of problematic use, frequency of checking and time spent on SNSs from approach and avoidance tendencies.

Variable	*R*^2^ change	*F* change	β	*p*
	Time spent using			
Step 1				
Approach	.004	*F*(1, 387) = 1.69	-.066	.195
Step 2				
Avoidance	.000	*F*(1, 386) = 0.17	-.026	.681
	Frequency of checking			
Step 1				
Approach	.016	*F*(1, 388) = 6.38	-.127	.012
Step 2				
Avoidance	.000	*F*(1, 387) = 0.02	-.008	.902
	Problematic use (SMDS score)			
Step 1				
Approach	.002	*F*(1, 376) = 0.65	-.042	.419
Step 2				
Avoidance	.000	*F*(1, 375) = 0.00	-.002	.979

#### Cue reactivity

Cue reactivity scores were not significantly correlated with either time spent using SNSs (*r* = .03, *p* = .582) or problematic use (*r* = .08, *p* = .096). However, cue reactivity scores were significantly correlated with frequency of checking (*r*_*S*_ = .21, *p* < .001) suggesting that individuals who are more reactive to SNS logos are more likely to check SNSs more frequently.

Furthermore, cue reactivity scores were significantly correlated with wanting scores (*r* = .21, *p* < .001) but not liking scores (*r* = .07, *p* = .134), confirming that cue reactivity to SNS logos is associated with urges/cravings to use SNS and not the pleasure experienced from using SNSs.

#### Wanting vs. liking

Wanting and liking scores were significantly correlated with each other (*r* = .38, *p* < .001). However, to assess the unique contribution of wanting above and beyond liking in predicting each of our SNS use measures we used a two-stage hierarchical regression analysis with liking scores entered at stage one and wanting scores entered at stage two (see Table 2). Liking scores significantly predicted time spent using SNSs, explaining 5.3% of the variance. However, the inclusion of wanting scores made a significant contribution to the model, explaining a further 2.1% of the variance. Nevertheless, liking scores (β = .171, *p* = .001) still provided the strongest contribution to the model after the inclusion of wanting scores. Liking scores also significantly predicted frequency of checking, explaining 6% of the variance. However, the inclusion of wanting scores explained 12.9% more of the variance and weakened the contribution of liking scores to the model (β = .097, *p* = .045). Similarly, liking scores predicted SMDS scores, explaining 5.4% of the variance. However, the inclusion of wanting scores explained 12.4% more of the variance and made the contribution of liking scores nonsignificant (β = .088, *p* = .076).

**Table 2:** Summary of regression analyses for the prediction of problematic use, frequency of checking and time spent on SNSs from wanting when controlling for liking.

Variable	*R*^2^ change	*F* change	β	*p*
	Time spent using			
Step 1				
Liking	.053	*F*(1, 408) = 22.96	.231	< .001
Step 2				
Wanting	.021	*F*(1, 407) = 9.21	.156	.003
	Frequency of checking			
Step 1				
Liking	.060	*F*(1, 409) = 26.00	.244	< .001
Step 2				
Wanting	.129	*F*(1, 408) = 65.04	.389	< .001
	Problematic use (SMDS score)			
Step 1				
Liking	.054	*F*(1, 396) = 22.51	.232	< .001
Step 2				
Wanting	.124	*F*(1, 395) = 59.55	.380	< .001

These results suggest that the experience of urges to use SNSs is a strong predictor of excessive checking and problematic use above and beyond the perceived enjoyment of using SNSs. They also suggest that the actual duration a user spends on SNSs is better predicted by how much they like the SNS rather than by urges or cravings.

### Exploratory analysis

Exploratory correlations were conducted between secondary variables (expected likes, money-or-likes and general sensitivity to reward) and each of our three outcome measures (time spent using, frequency of checking and problematic use). Results of these correlations are displayed in Table 3. We found that the number of “likes” an individual expects to receive on a typical post and the extent to which they would prefer to double the “likes” on a future post rather than receive money, were both positively correlated with frequency of checking. General sensitivity to reward was positively correlated with time spent on SNSs and problematic use but showed the strongest correlation with problematic use. Thus, individuals with increased sensitivity to the experience of reward may be more at risk of using SNSs problematically.

**Table 3:** Associations between secondary measures and the three usage variables.

	Time spent on SNSs	Frequency of checking	Problematic Use
Expected likes (N = 378)	*r* = .03, *p* = .629^a^	***r***_***s***_ = .18, *p* < .001*	*r* = .03, *p =* .614^b^
Money-or-likes (N = 400)	*r*_*s*_ = .04, *p* = .383^c^	***r***_***s***_ **= .14, *p* = .005**	*r*_*s*_ = .03, *p =* .535^d^
**GSR score (N = 370)**	***r* = .12, *p* = .020^e^**	***r*_*s*_ = .09, *p* = .089**	***r* = .20, *p* < .001** * **^f^**

*Note*. GSR score refers to general sensitivity to reward score. Non-parametric correlation analysis (Spearman’s Rho, *r*_*s*_) was used for *frequency of checking* and *money-or-likes* for which interval scaling could not be assumed.

* Indicates significant correlations that survive Bonferroni corrections for multiple tests.

^a^ N = 377.

^b^ N = 366.

^c^ N = 399.

^d^ N = 389.

^e^ N = 369.

^f^ N = 365.

## Discussion

The present study sought to identify implicit and explicit reward responses related to social networking sites (SNSs) in young adults and whether these are associated with use intensity and problematic use (social media addiction). We found that SNS logos elicited strong implicit approach motivation relative to control stimuli. Implicit approach motivation was also positively associated with the frequency of checking SNSs but not with addictive SNS use, which partially supported our first hypothesis.

Similarly, our second hypothesis was also partially supported as SNS cue reactivity scores were correlated with more frequent checking, but not with problematic use. Finally, partial support was found for our third hypothesis as although self-reported liking showed significant positive relationships with measures of excessive and problematic use, self-reported wanting explained a greater proportion of the variance in frequency of checking and problematic use above and beyond liking.

The clear presence of implicit approach tendencies in SNS users regardless of clinically relevant functional impairment (i.e., no association with social media addiction scores) signifies the special motivational status that social media have acquired in young adults’ lives. Social media app icons appear to have become potent reinforcers that are imbued with reward to the point of modulating basic processes of approach vs avoidance. The pervasive attractiveness of SNSs and their ability to elicit automatic approach responses is comparable to effects elicited by more basic rewards, such as food or sexual stimuli [47, 48]. Our previous survey results showed that SNS use is best understood as a behaviour motivated by the prospect of gaining social reward [46]. Specifically, we showed that problematic use and checking frequency were correlated with the motivation to obtain social approval from others, to make social comparisons or to mitigate fears that others have rewarding experiences without one’s own presence (Fear of Missing Out; [49]). The present results suggest that despite their seemingly more complex nature, these social rewards may be associated with neurocognitive mechanisms, such as automatic approach motivation, that are similar to those underpinning responses to primary rewards, including food and sexual stimuli.

Interestingly, the present data indicate that the control stimuli used in the approach-avoidance task elicited significantly faster avoidance responses compared to approach responses, suggesting a possible “avoidance bias” for these stimuli. We opted to use common non-SNS iPhone app icons as control stimuli since they share similar visual properties to SNS app icons, and we expected them to be relatively neutral with regard to their rewarding/aversive associations. However, if our control stimuli were truly neutral, we would expect no significant difference between approach and avoidance RTs to these images. It is thus possible that in regular SNS users these app icons have become devalued and are perceived as “less interesting” compared to the SNS logos being associated with reward and approach.

To our knowledge only two other studies have investigated automatic approach tendencies to SNS stimuli [33, 36]. Du et al. [36] found speeded approach RTs to Facebook stimuli in the whole sample, which were unrelated to social media self-control failures. These results were thus in line with the present findings and our interpretation that–similar to primary rewards–SNSs have high motivational salience in young adults. While Juergensen and Leckfor [33] did find a relationship between approach responses and Facebook addiction, effect sizes were small and significant correlations were found for only two of the six subscales used to assess addictive behaviours. Taken together, existing research in this area is limited and contains many methodological differences. Thus, it is currently too early to draw firm conclusions about the utility of implicit approach paradigms as markers of problematic SNS use.

Importantly, we found problematic use was predicted by explicit measures of SNS reward. Self-reported urges to use SNSs (= wanting) explained a significant proportion of the variance in both frequency of checking and problematic use. Pointing to a special role and possible dissociation of motivational (wanting) versus pleasure-related (liking) aspects of SNS use, wanting explained problematic use above and beyond self-reported liking (see also [8]). However, in contrast to our previous study, in the present study liking played a more important role in explaining the actual SNS usage duration. Additionally, the measure of cue reactivity produced a different pattern of results to those in our previous study [8]. While we replicated the finding that cue reactivity to SNS logos was correlated with more frequent checking, no associations were found with the usage duration and problematic use variables. However, a notable difference in the present study is that non-SNS logos were used as a baseline measure of general cue reactivity. Thus, when accounting for trait levels of reactivity to cues in general, the relationship between SNS cue reactivity and problematic/excessive use appears to be less important. We also found a significant positive correlation between general sensitivity to reward (measured with the SPSRQ) and problematic SNS use. This suggests that, similar to other addictions [50], the experience of greater reward sensitivity may represent a risk factor in the development of problematic SNS use behaviours [51]. Exploratory correlations also revealed that individuals who expect to receive a greater number of “likes” on their SNS posts and those who prefer to receive “likes” over money are more likely to check their accounts more frequently. This illustrates the powerful role that SNS “likes” might play in facilitating compulsive checking [cf. 52].

The present study is not without some important limitations. Firstly, the cross-sectional design does not permit us to make causal inferences about the direction of the reported effects, for instance whether frequent SNS checking produces an approach bias to SNS cues or whether pre-existing approach tendencies increase checking frequency. We also note the need to further control the stimuli used in the social media VAAST. The present study used non-SNS iPhone app icons as control stimuli, however these stimuli might not have been motivationally ‘neutral’ for all participants. Post-experimental stimulus ratings (e.g., on valence and motivational dimensions) may help to address this issue. Further, it is possible that the SNS logos were more familiar than control logos to participants. While differences in familiarity cannot account for the diverging pattern of RTs in approach vs avoidance trials in the present study, it might be useful to add familiarity ratings to future research. It is also notable that participants made significantly more errors on SNS approach trials compared to control approach trials and thus faster SNS approach tendencies could potentially be attributed to less accurate responding on these trials, indicating a speed accuracy trade-off. However, such an account would not explain the significantly slower RTs on SNS avoidance trials compared to controls since accuracy did not differ between these two conditions. Moreover, while speed-accuracy trade-offs can be problematic in RT tasks that use response times as a performance indicator (e.g., as a reflection of stimulus recognisability), here we interpret RT variations as being driven by motivational differences. In fact, increased approach tendencies or biases could be predicted to produce both faster AND less accurate responding, indicating the prioritisation of reward engagement over stimulus processing. Finally, while we did observe a significant relationship between self-reported checking frequency and implicit SNS approach tendencies, the correlation was weak and explained less than 2% of the variance. More research is needed to clarify the strength of this relationship, especially when checking frequency is assessed objectively through device-based tracking apps.

In sum, our results indicate that SNS logos elicited faster approach reactions compared to control app logos. Unlike some previous research [33] and contrary to our predictions, the difference between these RTs were not associated with diagnostic criteria of problematic use. However, approach tendencies to SNS stimuli were significantly (albeit weakly) associated with self-reported SNS checking (i.e., participants with faster approach RTs to SNS compared to control stimuli reported more frequent SNS checking). Checking frequency was also positively associated with urges elicited by SNS logos, corroborating the idea that sensitisation to cues associated with SNS reward constitutes an important determinant of use behaviours [8]. One tentative interpretation of the present pattern of results is that frequent SNS use goes along with dose-dependent changes in basic reward processes, including increases in automatic approach and use desires when confronted with SNS cues. However, for frequent checking to develop into social media addiction (i.e., continued use despite harmful consequences) other processes need to be present. These may include the subjective experience of SNS craving (wanting) regardless of the presence of cues but also the presence of pre-disposing trait variables, such as general reward sensitivity or impulsivity (lack of inhibitory control). Evidence for the role of inhibitory control comes from a study by Wegmann et al. [53]. Using an auditory Go/No-Go task with SNS ringtones (e.g., WhatsApp message tone) and analogous control stimuli (e.g., a bike bell) the authors showed that symptoms of disordered SNS use were highest in individuals who exhibited both weaknesses on measures of general attentional impulsivity and task-related inhibitory control (reacting to SNS ringtones when instructed to withhold responding). In another study, Gao et al. [54] employed a Go/No-Go task with SNS logos and control stimuli whilst also recording neural activity using an electroencephalogram (EEG). Although the behavioural data revealed no significant difference between excessive and non-excessive SNS users, EEG recordings revealed event related potentials (ERPs) indicative of inhibitory control difficulties in excessive compared to non-excessive SNS users. In a similar study using Facebook-related and affective images Moretta and Buodo [5] observed that problematic Facebook users displayed reduced accuracy to Go and No-Go trials regardless of image type and ERPs suggesting less efficient inhibitory control to natural and Facebook-related rewards (as indicated by reduced P3 amplitudes on No-Go trials).

To conclude, our study highlights an association between self-reported excessive checking of SNSs on the one hand and automatic approach tendencies and responses to SNS cues on the other hand. It also suggests altered explicit reward experiences in users displaying addictive use behaviours, including heightened SNS craving and amplified general reward sensitivity. At the same time, our study shows that more research employing experimental paradigms with SNS stimuli, such as the approach-avoidance or Go/No-Go task, is needed before inferences can be made about the similarities in the cognitive/motivational underpinnings of substance use addictions and problematic SNS use. The approach-avoidance task has previously been successfully implemented as a training tool to help reduce the consumption of addictive substances, including alcohol and tobacco [55]. Therefore, future studies might also assess whether approach bias modification to SNS cues can be used as an effective tool to reduce excessive SNS checking and thus potentially interrupt the pathway into social media addiction.
